# Supplementary material for: Meta-Analysis and Gene Set Enrichment Relative to ER Status Reveal Elevated Activity of MYC and E2F in the “Basal” Breast Cancer Subgroup
Source: PLoS One. 2009 Mar 9;4(3):e4710. doi: 10.1371/journal.pone.0004710 (PMC2650420; doi:10.1371/journal.pone.0004710)
Supplement: Dataset S1 — GSEA results from first screen. The file contains a zipped archive of the HTML results of the second GSEA screen. Reports for enriched or depleted datasets may be accessed from the “index.html” link within the my_analysis.GseaPreranked.1219975950625.rpt.zip ZIP archive. Enrichment plots are available for each of the 50 most enriched and depleted gene sets. (13.32 MB ZIP) [file pone.0004710.s001.zip › my_analysis.GseaPreranked.1219975950625.rpt.zip!//HDACI_COLON_TSA_UP.html]

Details for gene set HDACI\_COLON\_TSA\_UP[GSEA]

|  || Dataset | zSco.ERneg.to.ERpos\_collapsed |
| Phenotype | NoPhenotypeAvailable |
| Upregulated in class | na\_neg |
| GeneSet | HDACI\_COLON\_TSA\_UP |
| Enrichment Score (ES) | -0.38011006 |
| Normalized Enrichment Score (NES) | -1.7537551 |
| Nominal p-value | 0.0 |
| FDR q-value | 0.046983685 |
| FWER p-Value | 0.889 |
Table: GSEA Results Summary

  

Fig 1: Enrichment plot: HDACI\_COLON\_TSA\_UP      
 Profile of the Running ES Score & Positions of GeneSet Members on the Rank Ordered List

  

| PROBE | GENE SYMBOL | GENE\_TITLE | RANK IN GENE LIST | RANK METRIC SCORE | RUNNING ES | CORE ENRICHMENT || 1 | VGLL1 |  |  | 18 | 6.865 | 0.0318 | No |
| 2 | LRP8 |  |  | 163 | 5.498 | 0.0475 | No |
| 3 | SAT1 |  |  | 514 | 4.322 | 0.0419 | No |
| 4 | FBN2 |  |  | 862 | 3.683 | 0.0335 | No |
| 5 | ETV5 |  |  | 1073 | 3.387 | 0.0340 | No |
| 6 | COL4A3 |  |  | 1335 | 3.098 | 0.0292 | No |
| 7 | PLCB4 |  |  | 1692 | 2.765 | 0.0156 | No |
| 8 | FKBP5 |  |  | 1729 | 2.743 | 0.0262 | No |
| 9 | USP34 |  |  | 1807 | 2.686 | 0.0333 | No |
| 10 | SAMD4A |  |  | 2082 | 2.462 | 0.0245 | No |
| 11 | MT1H |  |  | 2140 | 2.422 | 0.0319 | No |
| 12 | SERPINA1 |  |  | 2175 | 2.403 | 0.0410 | No |
| 13 | PLAG1 |  |  | 2201 | 2.387 | 0.0506 | No |
| 14 | LUZP2 |  |  | 2509 | 2.196 | 0.0380 | No |
| 15 | KCNA1 |  |  | 2559 | 2.169 | 0.0448 | No |
| 16 | GNL1 |  |  | 3040 | 1.913 | 0.0177 | No |
| 17 | MBNL2 |  |  | 3321 | 1.785 | 0.0052 | No |
| 18 | C9ORF86 |  |  | 3333 | 1.779 | 0.0129 | No |
| 19 | FZD8 |  |  | 3705 | 1.619 | -0.0073 | No |
| 20 | CDKN1C |  |  | 3739 | 1.609 | -0.0020 | No |
| 21 | CYB5B |  |  | 3763 | 1.599 | 0.0040 | No |
| 22 | MTFR1 |  |  | 3803 | 1.587 | 0.0087 | No |
| 23 | VEGF |  |  | 3911 | 1.549 | 0.0081 | No |
| 24 | GPX3 |  |  | 4579 | 1.295 | -0.0361 | No |
| 25 | API5 |  |  | 4676 | 1.260 | -0.0373 | No |
| 26 | SLC2A9 |  |  | 4684 | 1.258 | -0.0317 | No |
| 27 | MAP1LC3B |  |  | 4713 | 1.249 | -0.0278 | No |
| 28 | AVPR1A |  |  | 4755 | 1.235 | -0.0249 | No |
| 29 | HTF9C |  |  | 4986 | 1.161 | -0.0367 | No |
| 30 | TRAM2 |  |  | 5311 | 1.043 | -0.0562 | No |
| 31 | PHKA1 |  |  | 5457 | 0.991 | -0.0624 | No |
| 32 | NEUROD6 |  |  | 5581 | 0.949 | -0.0671 | No |
| 33 | RPS3 |  |  | 5994 | 0.814 | -0.0943 | No |
| 34 | SF3A2 |  |  | 6044 | 0.796 | -0.0941 | No |
| 35 | BIRC4 |  |  | 6051 | 0.794 | -0.0908 | No |
| 36 | ODZ3 |  |  | 6070 | 0.791 | -0.0883 | No |
| 37 | EPB41 |  |  | 6428 | 0.672 | -0.1121 | No |
| 38 | C9ORF74 |  |  | 6593 | 0.624 | -0.1214 | No |
| 39 | IL10RA |  |  | 6598 | 0.621 | -0.1187 | No |
| 40 | JARID1C |  |  | 6662 | 0.602 | -0.1206 | No |
| 41 | ROCK1 |  |  | 6704 | 0.585 | -0.1209 | No |
| 42 | PC |  |  | 6791 | 0.556 | -0.1247 | No |
| 43 | ISG15 |  |  | 6857 | 0.533 | -0.1270 | No |
| 44 | HSF1 |  |  | 7062 | 0.463 | -0.1402 | No |
| 45 | TPM3 |  |  | 7148 | 0.436 | -0.1445 | No |
| 46 | INPP5E |  |  | 7393 | 0.347 | -0.1613 | No |
| 47 | ZUBR1 |  |  | 7541 | 0.291 | -0.1710 | No |
| 48 | AQP1 |  |  | 7776 | 0.199 | -0.1878 | No |
| 49 | CREBBP |  |  | 7868 | 0.158 | -0.1939 | No |
| 50 | GMCL1 |  |  | 8289 | 0.005 | -0.2256 | No |
| 51 | GLRX |  |  | 8308 | -0.001 | -0.2270 | No |
| 52 | HLA-DQB2 |  |  | 8357 | -0.020 | -0.2305 | No |
| 53 | SERPINE1 |  |  | 8947 | -0.283 | -0.2737 | No |
| 54 | ID2 |  |  | 9049 | -0.335 | -0.2797 | No |
| 55 | ZNF76 |  |  | 9135 | -0.376 | -0.2843 | No |
| 56 | CD36 |  |  | 9597 | -0.616 | -0.3162 | No |
| 57 | P4HB |  |  | 9771 | -0.707 | -0.3259 | No |
| 58 | C9ORF6 |  |  | 9807 | -0.729 | -0.3250 | No |
| 59 | ALG12 |  |  | 10349 | -1.060 | -0.3608 | No |
| 60 | PER1 |  |  | 10374 | -1.077 | -0.3574 | No |
| 61 | HSPA1A |  |  | 10471 | -1.135 | -0.3592 | No |
| 62 | C5 |  |  | 10561 | -1.215 | -0.3600 | No |
| 63 | CDH1 |  |  | 10717 | -1.328 | -0.3653 | No |
| 64 | MAP9 |  |  | 10770 | -1.366 | -0.3627 | No |
| 65 | RYBP |  |  | 10934 | -1.511 | -0.3677 | No |
| 66 | C7 |  |  | 10961 | -1.538 | -0.3622 | No |
| 67 | ZC3H12A |  |  | 10964 | -1.542 | -0.3549 | No |
| 68 | SLC20A1 |  |  | 11029 | -1.593 | -0.3521 | No |
| 69 | AHSA2 |  |  | 11114 | -1.678 | -0.3503 | No |
| 70 | GNAI2 |  |  | 11509 | -2.046 | -0.3702 | Yes |
| 71 | C14ORF122 |  |  | 11562 | -2.097 | -0.3640 | Yes |
| 72 | LASP1 |  |  | 11661 | -2.179 | -0.3609 | Yes |
| 73 | RBCK1 |  |  | 11676 | -2.197 | -0.3513 | Yes |
| 74 | LOC137886 |  |  | 11704 | -2.225 | -0.3426 | Yes |
| 75 | NEU1 |  |  | 11789 | -2.317 | -0.3378 | Yes |
| 76 | GRAMD1C |  |  | 11830 | -2.352 | -0.3294 | Yes |
| 77 | ECHDC2 |  |  | 12007 | -2.566 | -0.3303 | Yes |
| 78 | TSNAX |  |  | 12040 | -2.628 | -0.3200 | Yes |
| 79 | WIPI1 |  |  | 12116 | -2.722 | -0.3126 | Yes |
| 80 | NECAP1 |  |  | 12150 | -2.774 | -0.3017 | Yes |
| 81 | STX8 |  |  | 12209 | -2.850 | -0.2923 | Yes |
| 82 | CRYL1 |  |  | 12233 | -2.894 | -0.2800 | Yes |
| 83 | SCYL3 |  |  | 12331 | -2.998 | -0.2729 | Yes |
| 84 | FOSB |  |  | 12385 | -3.084 | -0.2620 | Yes |
| 85 | FOS |  |  | 12389 | -3.085 | -0.2473 | Yes |
| 86 | CDH11 |  |  | 12438 | -3.164 | -0.2356 | Yes |
| 87 | SDC4 |  |  | 12541 | -3.322 | -0.2273 | Yes |
| 88 | TRIM52 |  |  | 12737 | -3.709 | -0.2241 | Yes |
| 89 | FLNB |  |  | 12764 | -3.781 | -0.2078 | Yes |
| 90 | CALCOCO1 |  |  | 12786 | -3.834 | -0.1908 | Yes |
| 91 | PIP5K2C |  |  | 12796 | -3.883 | -0.1727 | Yes |
| 92 | COX6C |  |  | 12819 | -3.930 | -0.1554 | Yes |
| 93 | BCKDK |  |  | 12859 | -4.025 | -0.1389 | Yes |
| 94 | SPARCL1 |  |  | 12889 | -4.108 | -0.1212 | Yes |
| 95 | DAAM1 |  |  | 12945 | -4.313 | -0.1045 | Yes |
| 96 | RHOB |  |  | 13091 | -4.795 | -0.0923 | Yes |
| 97 | TNCRNA |  |  | 13139 | -4.984 | -0.0718 | Yes |
| 98 | GLI3 |  |  | 13213 | -5.527 | -0.0506 | Yes |
| 99 | RHBDF1 |  |  | 13243 | -5.792 | -0.0248 | Yes |
| 100 | KIAA0556 |  |  | 13280 | -6.318 | 0.0030 | Yes |
Table: GSEA details [plain text format]

  

Fig 2: HDACI\_COLON\_TSA\_UP: Random ES distribution      
 Gene set null distribution of ES for **HDACI\_COLON\_TSA\_UP**
